# Cephalosporinases associated with outer membrane vesicles released by *Bacteroides* spp. protect gut pathogens and commensals against β-lactam antibiotics

**DOI:** 10.1093/jac/dku466

**Published:** 2014-11-27

**Authors:** Régis Stentz, Nikki Horn, Kathryn Cross, Louise Salt, Charles Brearley, David M. Livermore, Simon R. Carding

**Affiliations:** 1 Gut Health and Food Safety Programme, Institute of Food Research, Norwich NR4 7UA, UK; 2 Analytical Sciences Unit, Institute of Food Research, Norwich NR4 7UA, UK; 3 School of Biological Sciences, The University of East Anglia, Norwich NR4 7TJ, UK; 4 Norwich Medical School, The University of East Anglia, Norwich NR4 7TJ, UK

**Keywords:** β-lactamases, protective effect, gut microbiota, *Salmonella*

## Abstract

**Objectives:**

To identify β-lactamase genes in gut commensal *Bacteroides* species and to assess the impact of these enzymes, when carried by outer membrane vesicles (OMVs), in protecting enteric pathogens and commensals.

**Methods:**

A deletion mutant of the putative class A β-lactamase gene (locus tag BT_4507) found in the genome of the human commensal *Bacteroides thetaiotaomicron* was constructed and a phenotypic analysis performed*.* A phylogenetic tree was built from an alignment of nine *Bacteroides* cephalosporinase protein sequences, using the maximum likelihood method. The rate of cefotaxime degradation after incubation with OMVs produced by different *Bacteroides* species was quantified using a disc susceptibility test. The resistance of *Salmonella* Typhimurium and *Bifidobacterium breve* to cefotaxime in liquid culture in the presence of *B. thetaiotaomicron* OMVs was evaluated by measuring bacterial growth.

**Results:**

The *B. thetaiotaomicron* BT_4507 gene encodes a β-lactamase related to the CepA cephalosporinase of *Bacteroides fragilis*. OMVs produced by *B. thetaiotaomicron* and several other *Bacteroides* species, except *Bacteroides ovatus*, carried surface-associated β-lactamases that could degrade cefotaxime. β-Lactamase-harbouring OMVs from *B. thetaiotaomicron* protected *Salmonella* Typhimurium and *B. breve* from an otherwise lethal dose of cefotaxime.

**Conclusions:**

The production of membrane vesicles carrying surface-associated β-lactamases by *Bacteroides* species, which constitute a major part of the human colonic microbiota, may protect commensal bacteria and enteric pathogens, such as *Salmonella* Typhimurium, against β-lactam antibiotics.

## Introduction

The adult human gastrointestinal (GI) tract accommodates a bacterial community (the microbiota) comprising trillions of cells that carry out vital functions for human health. This association involves co-evolved beneficial human–microbiota interactions that are altered as a result of many environmental factors. In particular, the presence of antibiotics can disturb colonic metabolism and absorption of vitamins, and can alter susceptibility to infection.^1,2^ The human GI tract microbiota is dominated by two bacterial phyla, Firmicutes and Bacteroidetes.^3^ Species of the genus *Bacteroides*, which constitute ∼30% of all bacteria in the human GI tract, are among the most resistant anaerobes to antibiotics,^4^ including penicillins and broad-spectrum cephalosporins.^5^

During the 1980s, β-lactamase-producing strains of *Bacteroides* species were reported to protect penicillin-susceptible β-haemolytic streptococci from penicillin G *in vivo*, as shown by monitoring the formation of subcutaneous abscesses in mice co-infected by both organisms.^6^ Likewise, β-lactamases produced by *Escherichia coli* can protect *Salmonella* Typhimurium when both organisms are co-cultured in liquid broth in the presence of ampicillin.^7^ It was suggested in this study that the β-lactamase protected the *Salmonella* against ampicillin once it was exported in outer membrane vesicles (OMVs) produced and released into the medium by the *E. coli* strain. Schaar *et al.*^8,9^ later confirmed this idea by demonstrating that β-lactamases associated with OMVs produced by *Moraxella catarrhalis* protect *Streptococcus pneumoniae* and *Haemophilus influenzae* against amoxicillin, and that OMVs produced by *H. influenzae* could protect group A streptococci against amoxicillin. The possibility that OMVs produced by commensal *Bacteroides* species possess similar capabilities has not been considered.

In this study, we describe a novel cephalosporinase gene in the genome of a prominent member of the human GI tract microbiota, *Bacteroides thetaiotaomicron*. Homologues of this gene exist in most, if not all *Bacteroides* species. We also describe the protective effect of β-lactamase-carrying OMVs produced by B. *thetaiotaomicron* on the susceptibility of *Salmonella* Typhimurium and *Bifidobacterium breve* to β-lactam antibiotics.

## Materials and methods

### Bacterial strains and growth conditions

*Bacteroides* species and strains (Table 1) were grown under anaerobic conditions at 37°C in brain heart infusion (BHI) medium (Oxoid/Thermo Fisher, Basingstoke, UK) supplemented with 0.5 mg/L haemin (Sigma-Aldrich, St Louis, MO, USA) (BHI–haemin). Antibiotic resistance markers in *B. thetaiotaomicron* were selected using 1 mg/L tetracycline, 5 mg/L erythromycin or 200 mg/L gentamicin. *E. coli* strains GC10 (Sigma-Aldrich) transformed with pGH014-based plasmids (see below) and HB101(pRK2013) (DMSZ Collection, Braunschweig, Germany) were grown under agitation at 37°C in LB medium^10^ supplemented with 100 mg/L spectinomycin and 50 mg/L kanamycin, respectively. Electrocompetent *E. coli* cells were prepared and transformed by the method of Sambrook and Russell (2001).^10^ *Salmonella enterica* serovar Typhimurium ATCC 14028 was grown at 37°C in LB medium under agitation.

**Table 1.** *Bacteroides* species and strains

Strain	Origin	Locus tag^a^	Combined disc method (mm)^c^	ESBL Etest^d^ (cefotaxime/cefotaxime plus clavulanic acid in mg/L)
*B. thetaiotaomicron* VPI-5482 (NCTC 10582, Werner, 1970)	DSMZ	BT_4507	14 ± 0.8	>16/1
*B. thetaiotaomicron* GH221	this work	ΔBT_4507	no difference	0.75/0.75
*B. dorei* DSM 17855	DSMZ	BACDOR_02757	9 ± 3.5	16/1
*B. fragilis* NCTC 9343 (Garrod, 1955)	DSMZ	BF_1199	12.6 ± 1.7	16/0.75
*B. ovatus* V975	Whitehead and Hespell^44^	BACOV975_02528^b^	18 ± 0.8	16/0.19
*B. stercoris* DMS 19555	DSMZ	BACSTE_01456	7 ± 0.8	>16/1
*B. xylanisolvens* XB1A ATCC 43183	DSMZ	GIB_3495	21.5 ± 0.5	>16/0.125

^a^The protein sequences were obtained from the National Center for Biotechnology Information (NCBI) protein databases.

^b^U. Wegmann, Institute of Food Research, Norwich, UK, personal communication.

^c^Difference between inhibition zones with cefpodoxime (10 μg) in the presence/absence of clavulanic acid (1 μg). The results shown are from three experiments performed independently.

^d^Etests were performed using strips containing cefotaxime and cefotaxime plus clavulanic acid.

### Electron microscopy

Samples were fixed for 1 h in 2.5% glutaraldehyde in 0.1 M piperazinediethanesulfonic acid (PIPES) buffer (pH 7.2). After washing with 0.1 M PIPES buffer, each sample was pipetted onto the centre of a small square of filter paper, which was folded and inserted into a critical-point drying capsule and dehydrated in a series of ethanol solutions (10, 20, 30, 40, 50, 60, 70, 80, 90 and 3 × 100%). Samples were critical-point dried in a Polaron E3000 drier (Quorum Technologies, Newhaven, UK) using liquid carbon dioxide as the transition fluid. The parcels were then carefully unfolded and the dry cells attached to sticky tabs mounted on scanning electron microscopy stubs by flicking the back of the filter paper in the direction of the stub. The samples were coated with gold in a high-resolution sputter-coater apparatus (Agar Scientific, Stansted, UK). Scanning electron microscopy was carried out using a Zeiss Supra 55 VP FEG SEM operating at 3 kV.

For negative staining, a drop of vesicle suspension was applied to a carbon-coated Formvar copper grid and left for 1 min before washing with five or six drops of 2% uranyl acetate solution in water. The excess stain was wicked off and the grids were left to dry thoroughly before viewing in the transmission electron microscope. The grids were examined and imaged in an Tecnai G2 20 Twin transmission electron microscope (FEI, Hillsboro, OR, USA) at 200 kV.

### Oligonucleotide primers

The primers used are detailed in Table S1 (available as Supplementary data at *JAC* Online).

### Construction of a BT_4507 deletion mutant

A 798 bp chromosomal DNA fragment upstream from BT_4507 and including the first 18 nucleotides of its 5′-end region was amplified by PCR using the primer pair BT4507_1 and BT4507_2. This product was then cloned into the SacI/BamHI site of the *E. coli*–*Bacteroides* suicide shuttle vector pGH014, consisting of plasmid pFD516^11^ with the tetracycline resistance gene *tetQ* from the *Bacteroides* plasmid pBT-2^12^ inserted into the BamHI and SalI restriction sites. A 829 bp chromosomal DNA fragment downstream from BT_4507, including the last 46 nucleotides of the 3′-end region, was amplified by PCR using the primer pair BT4507_3 and BT4507_4 and was cloned into the SalI/PstI site of the pGH014-based plasmid. The resulting plasmid, containing the BT_4507::*tetQ* construct, was mobilized from *E. coli* GC10 into *B. thetaiotaomicron* by triparental filter mating,^13^ using *E. coli* HB101(pRK2013) as the helper strain. Transconjugants were selected on BHI–haemin agar containing 200 mg/L gentamicin and 1 mg/L tetracycline. Determination of susceptibility to either tetracycline or erythromycin was carried out to identify recombinants that were tetracycline resistant and erythromycin susceptible, after re-streaking transconjugant bacteria on LB agar containing tetracycline or both antibiotics. PCR analysis and sequencing were used to confirm the allelic exchange. A transconjugant, GH221, containing the BT_4507::*tetQ* construct inserted into the *B. thetaiotaomicron* chromosome was selected for further studies.

### Overexpression of BT_4507 in the deletion mutant

To complement the *B. thetaiotaomicron* BT_4507 deletion mutant GH221, a derivative of the *Bacteroides* vector pGH043,^14^ was engineered for high-level expression of BT_4507. First, the primer pair Lactamase_F and Lactamase_EcoRI_R was used to amplify an 889 bp region encoding BT_4507 from *B. thetaiotaomicron* VPI-5482 genomic DNA. This BT_4507 fragment was digested with EcoRI before cloning into the NcoI (blunted)/EcoRI site of the *Bacteroides* expression vector pGH043, creating pGH092. To visualize the BT_4507 protein by fluorescence imaging, we made an in-frame fusion of the flavin mononucleotide-based fluorescent protein Pp1 with the C-terminus of BT_4507, using a 30-amino-acid linker. A PCR fragment of Pp1 was then obtained, using pGLOW-Pp1-stop (Evocatal GmbH, Dusseldorf, Germany) plasmid DNA as template. This fragment, attached to the linker by recombinant PCR, was cloned into the BsaAI/EcoRI site of pGH092, creating pGH095. To achieve overexpression, the ribosome-binding site region present in pGH095 was exchanged with the ribosome-binding site region of vector pGH090,^14^ by way of splice extension PCR. Initially, the primer pairs Reverse and 20-Lact_R, and 20-Lact_F and Lactamase_EcoRI_R were used, respectively, on templates pGH090 and pGH092. The resulting products were then used as templates for the splice PCR involving the primer pair Reverse and Lactamase_EcoRI_R. The 683 bp SphI, PshAI-digested PCR fragment was used to replace the corresponding 761 bp digest fragment of pGH095, creating pGH098. Transformation of the BT_4507 deletion mutant GH221 with pGH098 resulted in the creation of GH274.

### Phylogenetic analysis

The evolutionary relationship of β-lactamases was inferred using the maximum likelihood method in the MEGA5 software tool.^15^ Amino acid sequences were aligned with PRANK,^16^ and highly variable regions removed from the dataset. The Whelan and Goldman model of evolution^17^ was used and initial tree(s) for the heuristic search were obtained automatically as follows. When the number of common sites was <100, or otherwise less than one-fourth of the total number of sites, the maximum parsimony method was used; otherwise the BIONJ method,^18^ with maximum composite likelihood (MCL) distance matrix, was used.^19^ To provide statistical support for each node on the tree, a consensus tree was generated from 1000 bootstrap datasets.

### OMV isolation

Bacterial cultures (20 mL) were centrifuged at 5000 **g** for 15 min at 4°C and the supernatants were filtered through 0.22 μm pore-size polyethersulfone (PES) membranes (Sartorius, Goettingen, Germany) to remove debris and cells. Supernatants were concentrated by ultrafiltration (100 kDa molecular weight cut-off, Vivaspin 20, Sartorius) to 200 μL. The retentate was rinsed twice with 20 mL of PBS (pH 7.4) and concentrated to 200 μL (2 mg dry weight vesicles). OMV sterility was examined by checking for growth of any contaminating bacterial cells on BHI–haemin agar. OMV protein content was determined using the Total Protein Micro protein assay reagent kit (Sigma-Aldrich) after disruption by sonication. Alternatively, the dry weight of OMVs was determined after incubating OMV suspensions in a drying oven for 48 h. OMVs were also collected from the lower compartment of a Steritop filtration unit (Millipore, Billerica, USA) containing sterile BHI–haemin medium on a magnetic stir plate, after their diffusion through a 0.22 μm pore membrane from the upper compartment containing a growing *B. thetaiotaomicron* culture in BHI–haemin. The sterility of the BHI–haemin containing the vesicles was confirmed by plating OMV suspensions onto BHI–haemin agar immediately before collection.

### Measurement of β-lactamase activity

*Bacteroides* species were grown in 20 mL of BHI–haemin for 16 h, centrifuged at 3500 **g** for 10 min and the periplasmic fraction was prepared according to the method described by Osborn *et al*.^20^ Briefly, the cell pellet was resuspended in 4 mL of fractionation buffer (30 mM Tris, 20% sucrose, 1 mM EDTA, pH 8) and incubated for 10 min at 20°C, then centrifuged for 10 min at 3000 **g**. This pellet was resuspended in 0.8 mL of ice-cold 5 mM MgSO_4_ and left on ice for 10 min, releasing an osmotic shock fluid, which was separated by centrifugation for 10 min at 3000 **g**, 4°C. OMV concentrations were adjusted according to the protein concentrations obtained after sonication.

To assess the BtCepA β-lactamase activity, 5 μL of protein extract (corresponding to 5 μg for the vesicles) was added to 95 μL of phosphate buffer (0.1 M phosphate/1 mM EDTA, pH 7.0) and assayed spectrophotometrically at 486 nm by hydrolysis of 50 mg/L nitrocefin according to the manufacturer's instructions (Calbiochem).

### Enzyme activity at the surface of OMVs

A suspension of 250 μg of vesicles in 0.1 M phosphate/1 mM EDTA buffer (pH 7.0) was incubated for 5 min (for vesicles from *B. thetaiotaomicron*) and 1 h (for all other *Bacteroides* species) at 37°C in the presence of 100 mg/L proteinase K (Sigma-Aldrich). Proteinase K activity was stopped by addition of 1 mM phenylmethanesulfonyl fluoride (PMSF). To measure β-lactamase activity, 50 mg/L nitrocefin was added and changes in absorbance at 486 nm were measured. For BtMinpp activity, 1 mM inositol hexakisphosphate (InsP_6_) (Merck, Readington, NJ, USA) was added to the OMVs and the mix was incubated for 1 h at 37°C. Inositol phosphates were resolved by anion exchange chromatography as previously described.^21^ As a control, 25 mg/L of purified His-tagged BtMinpp was incubated^21^ with or without 100 mg/L proteinase K for 1 h at 37°C, with 1 mM PMSF added to stop the reaction. Degradation of 1 mM InsP_6_ was measured by HPLC (method described in Stentz *et al.*^21^) after incubation for 1 h at 37°C. BtMinpp activity was quantified as the ratio of the sum of the integrated peak areas of InsP_5_ products to the sum of the integrated InsP_5_ and InsP_6_ peaks.

### β-Lactamase activity in OMVs from multiple Bacteroides species

For OMV production, the different *Bacteroides* species were grown in the presence of 10 mg/L cefotaxime to ensure optimized cephalosporinase expression. Vesicles corresponding to 10 μg of total protein obtained by sonication were added to 100 μL of a 10 mg/L cefotaxime solution. The antibiotic solution containing OMVs was incubated for 1 h at 37°C, with OMVs subsequently removed by filtration (Amicon Ultra-0.5 centrifugal filter device, 100 kDa, Millipore, Billerica, MA, USA), and 10 μL of filtrate (corresponding to 0.1 μg of cefotaxime) was applied to a disc previously placed on a *Salmonella*-inoculated soft agar overlay plate. The inhibition zones were measured after 16 h of incubation at 37°C.

### Susceptibility tests

The susceptibility of *Bacteroides* to cefpodoxime and cefpodoxime plus clavulanic acid was tested using a cefpodoxime combination disc kit (Oxoid/Thermo Fisher, Basingstoke, UK) with the discs placed on BHI agar plates topped with a soft agar layer (0.75% agar) seeded with *Bacteroides* species. Double-ended Etest^®^ strips (AB bioMérieux, Marcy-l'Étoile, France) containing gradients of cefotaxime (16–0.25 mg/L) at one end and cefotaxime (1–0.016 mg/L) plus clavulanic acid at the other end were used to measure susceptibility, on BHI agar. The different *Bacteroides* species used were pre-grown in the presence of 5 mg/L cefotaxime and the cells were rinsed with fresh BHI broth.

### OMV–Salmonella and OMV–Bifidobacterium co-incubations

Cultures of *S. enterica* serovar Typhimurium strain ATCC 14028 and *B. breve* UCC2003,^22^ were grown for 16 h at 37°C under agitation in LB broth and anaerobically in BHI broth supplemented with 0.5% yeast extract (BHIY), respectively. Aliquots of 10 μL of 100-fold dilutions of these cultures were added to 10 mL of pre-warmed LB or BHIY, respectively. OMVs from a 20 mL culture of *B. thetaiotaomicron* (corresponding to 20 μg of total protein) were concentrated, rinsed and re-suspended in PBS. OMV concentrations were adjusted according to the protein concentrations measured after sonication. After addition of different concentrations of cefotaxime the *Salmonella* culture was incubated at 37°C with agitation (250 rpm) and the *Bifidobacterium* culture was incubated anaerobically while being stirred at 37°C. At varying times thereafter cells were plated for viable counts.

## Results

### BT_4507 encodes a β-lactamase that confers resistance to high levels of ampicillin

To determine whether OMVs produced by *B. thetaiotaomicron* harbour β-lactamase activity, we first identified the gene(s) responsible for the resistance of the bacterium to β-lactam antibiotics.

**Figure 1.**
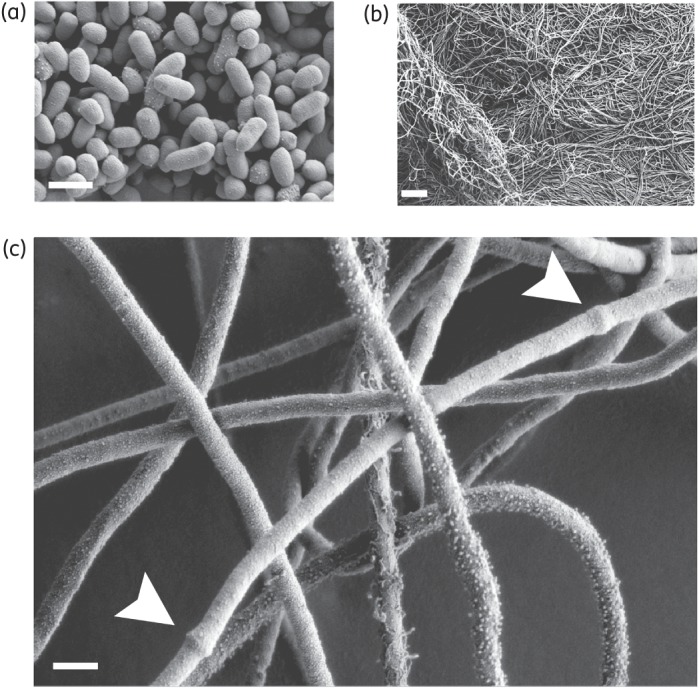
Scanning electron microscope image of *B. thetaiotaomicron* cells. (a) Non-treated cells. (b and c) Cells grown in the presence of 10 mg/L ampicillin. Scale bar, ∼10 μm (b) and 1 μm (a and c). The nodes, likely to represent the site of cell septation, are indicated with white arrows in (c).

We began by measuring the resistance of *B. thetaiotaomicron* VPI-5482 to penicillins, observing high-level resistance to the penicillin derivative ampicillin (Table 2). We also noted that *B. thetaiotaomicron* grown in the presence of sublethal concentrations of ampicillin (10–25 mg/L) gave rise to large numbers of tangled filaments >150 μm long (Figure 1b), comprising non-septate cells 12.5 μm in length. In considering the distance between two nodes (Figure 1c), representing the site of cell septation in normal conditions, this dimension is >10-fold greater than that of non-treated cells (Figure 1a). Examples of filament formation in *Bacteroides* species grown with subinhibitory concentrations of β-lactam antibiotics have previously been reported.^23^ For *E. coli* and other Gram-negative aerobes,^24,25^ the binding of β-lactam antibiotics to PBP3, a key element of the cell-septation machinery, prevents cell division and leads to the formation of filamentous cells.

**Table 2.** Resistance to ampicillin and related β-lactamase activity in *B. thetaiotaomicron*

Strain	Genotype^a^	MIC^b^	β-Lactamase activity^c^ in the periplasm	β-Lactamase activity^c^ associated with OMVs^d^		
				total activity	vesicle fraction	buffer
GH196	WT (pGH043)	32	269 ± 8	33 ± 6	29 ± 5	ND
GH266	ΔBT_4507 (pGH043)	1	0.1 ± 0.3	ND	ND	ND
GH274	ΔBT_4507 (pGH098)	1024	12 415 ± 551	284 ± 14	271 ± 11	2.3 ± 0.5

ND, not detected.

^a^pGH043, empty vector; pGH098, plasmid overexpressing BT_4507.

^b^Concentration of ampicillin in mg/L. The results are from two experiments performed independently.

^c^β-Lactamase activity expressed in nmol of hydrolysed nitrocefin/mg of protein/min. The activity was assessed spectrophotometrically by hydrolysis of nitrocefin. The results shown are from three experiments performed independently.

^d^The vesicles were incubated in phosphate buffer (0.1 M phosphate/1 mM EDTA, pH 7.0) for 1 h at 37°C and the total activity in the suspension was measured after sonication. Alternatively, the vesicles were removed by filtration and the activity was measured in the vesicle fraction and in the filtered buffer.

The genome of *B. thetaiotaomicron* VPI-5482 (NCTC 10582)^26^ contains four putative β-lactamase genes: three putative class B3 metallo-β-lactamase (MBL) genes (locus tags BT_0822, BT_1146 and BT_1410) and a class A serine β-lactamase gene (BT_4507). There is, however, a significant risk in naming these gene products as class B3 MBLs because of their sequence and structural homology to e.g. metallo-glyoxylases II (based on pairwise sequence alignments using BLASTP,^27^ and protein structure alignments performed with 3D-JIGSAW^28^).^29^ In addition, because of the susceptibility of the strain and the genus *Bacteroides* as a whole to carbapenems, we concluded that MBLs were not a significant factor in resistance and focused our attention on the serine β-lactamase. The BT_4507-encoded β-lactamase protein contains a predicted secretion signal (http://www.cbs.dtu.dk/services/SignalP/), implying that this enzyme is potentially secreted into the periplasm and could subsequently be packaged into OMVs, as shown for other bacterial species.^30^ We therefore chose to study BT_4507 and constructed a deletion mutant, which lost resistance to ampicillin, further supporting the absence of other significant β-lactamase activities (Table 2). For ease of detection of β-lactamase activity, a *B. thetaiotaomicron* strain was engineered to overexpress BT_4507. Overexpression of BT_4507 *in trans* in the deleted mutant raised the MIC of ampicillin to levels 32-fold higher than for the WT strain (Table 2). Moreover, the β-lactamase activities measured in periplasmic protein extracts of the different variants correlated with the level of resistance to ampicillin (Table 2).

### Identification of novel cepA-like chromosomal genes encoding cephalosporinases in Bacteroides species

**Figure 2.**
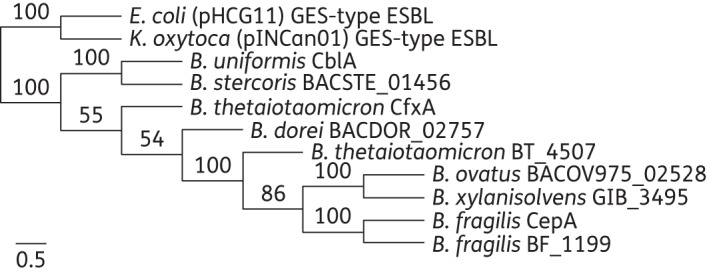
Phylogenetic tree derived from the alignment of 11 β-lactamase proteins from different bacterial species constructed using the maximum likelihood method. To provide statistical support for each node on the tree, a consensus tree was generated from 1000 bootstrap datasets. The tree is drawn to scale, with branch lengths measured as the number of substitutions per site. *E.*, *Escherichia*; *K.*, *Klebsiella*; *B.*, *Bacteroides*; GES enzymes are plasmid-mediated types that are disseminated among Enterobacteriaceae.

A BLASTP search of the BT_4507-encoded protein sequence against the non-redundant protein database^31^ identified 273 sequences with significant alignments (E value ≤1 e^−40^ and a minimal number of 60 identical amino acids over the entire sequence length). These protein sequences derived mainly from bacteria of the Bacteroidetes phylum. Among them, CepA and CfxA are two class-A cephalosporinase variants from *B. fragilis*^32,33^ and CblA is a class A cephalosporinase from *Bacteroides uniformis*.^34^ A phylogenetic tree was constructed by alignment of nine *Bacteroides* representatives (Figure 2), with two GES-type β-lactamases from Enterobacteriaceae as an outgroup.^35^ BT_4507 belonged to the CepA clade, which includes CepA from *B. fragilis.* We shall therefore refer to the BT_4507-encoded enzyme as BtCepA.

To test whether the cephalosporinase homologues from reference *Bacteroides* listed in the phylogenetic tree were β-lactamases, we adapted two phenotypic confirmatory tests developed for Enterobacteriaceae ESBLs to *Bacteroides*.^36^ Resistance to third-generation cephalosporin antibiotics was measured and compared in the presence and absence of clavulanic acid, which inhibits class A β-lactamases (Table 1). Except for the *B. thetaiotaomicron cepA* deletion mutant, the zone size measured in the combined disc method using cefpodoxime (10 μg) increased by >5 mm when clavulanic acid (1 μg) was added. This result was confirmed using a cefotaxime-based Etest method (Table 1). Therefore, all *Bacteroides* tested contained at least one class A cephalosporinase gene.

The cephalosporinase enzymes inferred to be the products of these genes were isolated, purified and extensively characterized from crude extracts of clinical isolates of *B. thetaiotaomicron*, *B. fragilis* and *Bacteroides vulgatus* by Sato *et al*.^37^ three decades ago. The susceptibility profile of *B. thetaiotaomicron* VPI-5482 is unexceptional for a *Bacteroides* species, with susceptibility to carbapenems, co-amoxiclav and cefoxitin (data not shown), but resistance to ampicillin, amoxicillin, cefotaxime and cefpodoxime.

### B. thetaiotaomicron OMVs are naturally produced and carry BtCepA on their surface

**Figure 3.**
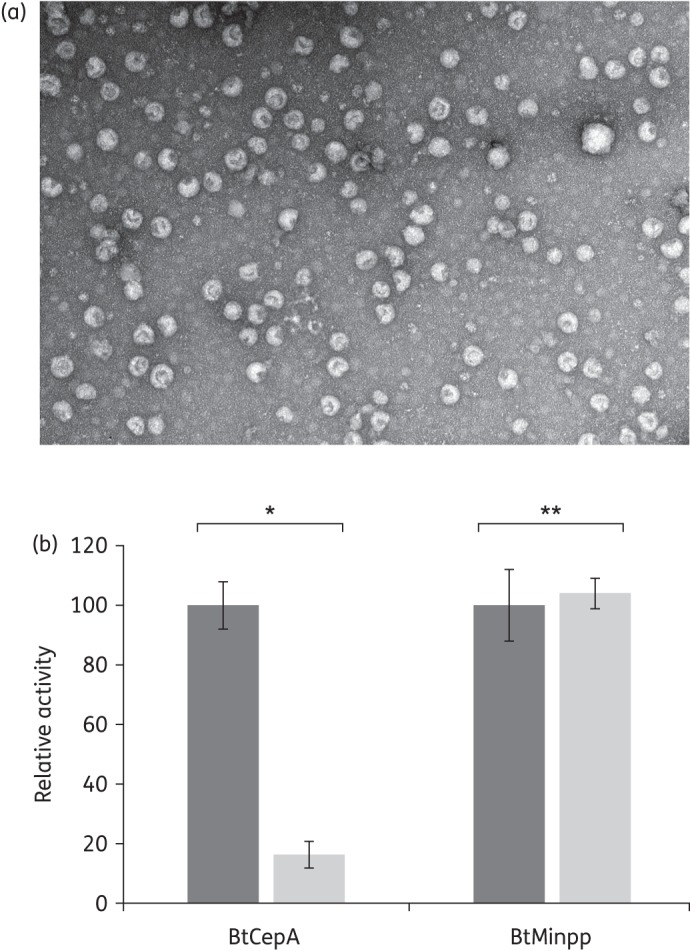
OMVs are produced *in vivo* by *B. thetaiotaomicron* and display BtCepA on their surface. (a) Electron microscopic photograph of OMVs collected from a sterile compartment after their diffusion through a 0.22 μm membrane from a compartment containing a *B. thetaiotaomicron* culture (see the Materials and methods section). Scale bar, ∼100 nm. (b) BtCepA and BtMinpp activities measured after treatment of OMVs with proteinase K. The relative activity is the ratio of the activity measured after proteinase K treatment compared with the activity measured without treatment. Dark grey bars, no proteinase K pre-treatment; light grey bars, proteinase K pre-treatment. *P < 0.0001; **P = 0.69.

We recently established that the phosphatase BtMinpp is exported and active in OMVs produced by *B. thetaiotaomicron*.^21^ However, one of the first steps of the standard protocol used for OMV isolation involves centrifugation of a bacterial culture in order to separate bacterial cells from the soluble fraction containing the vesicles. This centrifugation can cause cell surface damage, giving a potential for artefacts.^38^ Therefore, to ensure that OMVs isolated after cell centrifugation are produced during bacterial cell growth and are not derived from ruptured and damaged cells, we developed an alternative procedure in which OMVs were collected from a growing bacterial culture after their diffusion through a membrane filter into a compartment containing sterile medium (see the Materials and methods section). The OMVs obtained were visualized by electron microscopy (Figure 3a) and were indistinguishable from those generated by the standard centrifugation protocol, confirming that they are produced by living cells and not by, e.g. centrifugal shear forces.

We next addressed whether BtCepA is exported to OMVs. Assessment of the β-lactamase activity in *B. thetaiotaomicron* OMV protein extracts confirmed that BtCepA is associated with OMVs (Table 2). To localize BtCepA, OMV preparations were treated with proteinase K to digest any BtCepA associated with the outer surface of the vesicles. Only 5 min of proteinase K treatment was required for OMVs to lose 84% of their enzymatic activity when compared with non-treated OMVs (Figure 3b). Control experiments showed that activity of the BtMinpp phosphatase was not affected by proteinase K, confirming its location inside the vesicles. This result strongly suggests that a large fraction of BtCepA is exposed on the surface of the vesicles.

To ensure that the β-lactamase activity measured in solutions containing OMVs was not the result of enzyme release from OMVs into the milieu, a control experiment was run using vesicles isolated from *B. thetaiotaomicron* over-expressing BtCepA. No activity was detected in the milieu after 1 h of incubation (Table 2), consistent with the degradation of exogenous β-lactam antibiotics by OMVs occurring primarily at their surface and to a lesser extent within the vesicles.

### Cephalosporinases are exported on the surface of OMVs released by other Bacteroides species

**Figure 4.**
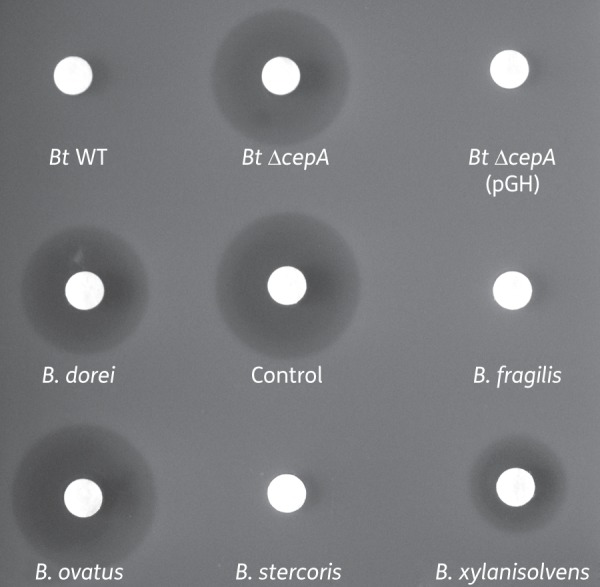
OMVs produced by *Bacteroides* spp. degrade cefotaxime. Antibiotic disc susceptibility test using *Salmonella* Typhimurium. The discs were loaded with 10 μL of a 10 mg/L cefotaxime solution that had been incubated for 1 h at 37°C with OMVs from different *Bacteroides* species. The discs were then placed onto *Salmonella*-inoculated agar plates. The inhibition zones were read after 16 h of incubation at 37°C. Control, 0.1 μg cefotaxime.

Based on the results obtained with BtCepA, we anticipated that cephalosporinases from other *Bacteroides* species would also be exported to OMVs. Accordingly, we investigated the capacity for isolated vesicles from *Bacteroides dorei*, *B. fragilis*, *Bacteroides ovatus*, *Bacteroides stercoris* and *Bacteroides xylanisolvens* to degrade β-lactam antibiotics present in the external milieu. OMVs were incubated in the presence of the third-generation cephalosporin cefotaxime and the degradation of the antibiotic was measured by applying the antibiotic solution, once OMVs had been removed, to a blank filter paper disc on a plate seeded with *Salmonella* as the indicator organism (Figure 4). *S. enterica* serovar Typhimurium has previously been used as an indicator strain in studies showing a protective effect by ampicillin-resistant *E. coli*.^7^

OMVs from *B. fragilis*, *B. stercoris* and *B. thetaiotaomicron* showed the highest capacity to degrade cefotaxime, with no visible inhibition of *Salmonella* growth remaining (Figure 4). In contrast, a zone of inhibition similar to that with untreated antibiotic was observed with *B. ovatus* OMVs, indicating that no β-lactamase activity was present in/on these OMVs despite expression of an active cephalosporinase (Table 1). These data suggest that, under laboratory conditions, the cephalosporinase produced by *B. ovatus* may be excluded from OMVs, as previously shown for various outer membrane proteins in *E. coli*,^30^ or, alternatively, does not bind to the surface of OMVs.

Using the proteinase K procedure applied to *B. thetaiotaomicron* OMVs (see above), 80%, 86% and 93% of the β-lactamase activity is exposed on the surface of *B. fragilis*, *B. stercoris* and *B. xylanisolvens* OMVs, respectively. The activity from *B. dorei* OMVs was very weak, making evaluation of the surface-exposed fraction difficult.

The clear reduction in the cefotaxime inhibition zone following exposure to OMVs from most *Bacteroides* species strongly suggests that these OMVs have the capability to serve as antibiotic degradation machineries in the GI tract of animals, acting remotely and independently of their parental bacterial cells.

### B. thetaiotaomicron OMVs protect Salmonella Typhimurium and B. breve against β-lactam antibiotics

**Figure 5.**
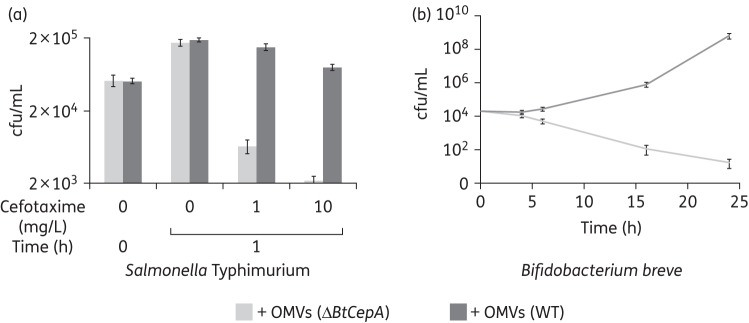
Killing curves of *Salmonella* Typhimurium (a) and *B. breve* (b) in liquid broth in the presence of *B. thetaiotaomicron* OMVs produced either by the Δ*BtcepA* mutant (light grey) or the WT strain (dark grey). The final concentration of cefotaxime added to the culture at time zero is indicated.

To confirm that OMVs carrying cephalosporinases can protect bacteria from β-lactam activity, *B. thetaiotaomicron* OMVs were added to growing cultures of *Salmonella* Typhimurium (Figure 5a) or to those of the human commensal intestinal bacterium *B. breve* (Figure 5b) in the presence or absence of cefotaxime. Vesicles produced by the Δ*BtcepA* deletion mutant were used as a negative control. As expected, cefotaxime had a dramatic effect on the viability of *Salmonella* Typhimurium cells when grown in the presence of vesicles lacking *BtcepA* (Figure 5a). By contrast, addition of BtCepA-loaded vesicles allowed the salmonellae to grow normally in the presence of the lowest cefotaxime concentration (1 mg/L) and at a slower rate for the highest concentration (10 mg/L). The same phenomenon was observed when ampicillin was used (data not shown). Because of its slower growth rate, *B. breve* appeared more resistant to cefotaxime than *Salmonella* Typhimurium over time (Figure 5b). However, the presence of *B. thetaiotaomicron* BtCepA-loaded vesicles in the medium allowed the cells to grow until saturation of the culture at 24 h, whereas most of the cells were killed after 24 h when the culture was supplemented with ΔBtcepA vesicles. Thus, β-lactamase-loaded OMVs from *Bacteroides* protected *Salmonella* Typhimurium and *B. breve* against β-lactam antibiotics.

## Discussion

Our study focuses on the identification of cephalosporinase genes in human gut *Bacteroides* species and on the distribution of their product. We find that, in addition to being secreted to the periplasm of bacterial cells, these cephalosporinases also associate with OMVs produced by these bacteria; the results are consistent with a role for these OMVs in degrading β-lactam antibiotics remotely from their parental cells.

Whereas Gram-positive β-lactamase-producing organisms largely liberate their enzymes into the surrounding milieu, the β-lactamases of Gram-negative bacteria are usually considered to be confined to the periplasmic space of the cells, acting in concert with the diffusion barrier of the outer membrane.^39^ The outer membrane of *E. coli* constitutes a diffusion barrier for both penicillins and cephalosporins;^40^ it thereby slows the influx of antibiotic molecules and is one of the major factors that determine the degree of antibiotic resistance of the organism.^41^ In this report, we show that part of cephalosporinase BtCepA becomes associated with membrane vesicles and this BtCepA–OMV association efficiently degrades substrate β-lactam antibiotics present in the surrounding medium. The extracellular degradation of β-lactam antibiotics is attributable to the OMVs only, since no leakage of the enzyme into the medium could be detected (Table 2). Degradation experiments with proteinase K showed that BtCepA was exposed on the surface of the vesicles rather than being sequestered within their lumen (Figure 3b). This is of interest since surface-exposed enzymes, exposed to the full antibiotic concentration, can achieve swifter substrate turnover than periplasmic enzymes in hydrolysing β-lactams in the surrounding medium^40^ and, by extension, would be more efficient than enzymes located inside OMVs.

Although our findings are consistent with a large fraction of BtCepA being associated with the membrane of OMVs and being exposed on the surface of the vesicles, no transmembrane region was predicted in BtCepA using the TMHMM server (http://www.cbs.dtu.dk/services/TMHMM-2.0/), suggesting that BtCepA is unlikely to be embedded in the outer membrane. It is also unlikely that BtCepA is a lipoprotein, since *Bacteroides* β-lactamases give discrete and sharp bands by isoelectric focusing analysis,^42^ instead of smearing, as expected for lipoproteins. BtCepA and other *Bacteroides* cephalosporinases perhaps may electrostatically associate with surface phosphates of vesicle phospholipids, but further analysis of their exact localization and attachment is needed, in both bacterial cells and OMVs, to confirm or refute this hypothesis and to determine exactly how these proteins anchor to the surface of OMVs.

It is well established that antibiotic usage strongly affects the intestinal microbiota.^2,43^ For example, Pérez-Cobas *et al.*^2^ recently reported that combined intravenous therapy with ampicillin/sulbactam and cefazolin caused significant microbiota disturbance in the human colon. Hence, we speculate that *Bacteroides* species in the human GI tract microbiota have the potential to protect *Salmonella* and possibly other pathogens and commensal microorganisms, such as the probiotic *B. breve*, by yielding large numbers of cephalosporinase-coated OMVs. More generally, the presence of cephalosporinase-coated OMVs in the colon could contribute to the maintenance of a balanced microbiota protecting against the adverse effects of antibiotic treatments. It remains unclear whether members of the genus have specifically evolved to do this during the antibiotic era or whether, as seems more likely, the association of these cephalosporins, which are inherent to most members of the genus, and the OMVs is essentially fortuitous. Notably, the *B. fragilis* NCTC 9343 strain, which produces β-lactamase-coated vesicles, was deposited with a culture collection as early as 1955, suggesting that the behaviour is not a very recent development.

## Funding

This work was supported by institutional grants from the Biotechnology and Biological Sciences Research Council (BBSRC) (BB/J004529/1, S. R. C.).

## Transparency declarations

None to declare.

## Supplementary data

Table S1 is available as Supplementary data at *JAC* Online (http://jac.oxfordjournals.org).

Supplementary Data
