# Supplementary material for: Cephalosporinases associated with outer membrane vesicles released by Bacteroides spp. protect gut pathogens and commensals against β-lactam antibiotics
Source: J Antimicrob Chemother. 2014 Nov 27;70(3):701–9. doi: 10.1093/jac/dku466 (PMC4319488; doi:10.1093/jac/dku466)
Supplement: Supplementary Data [file supp_70_3_701__index.html]

Cephalosporinases associated with outer membrane vesicles released by Bacteroides spp. protect gut pathogens and commensals against β-lactam antibiotics — Cephalosporinases associated with outer membrane vesicles released by Bacteroides spp. protect gut pathogens and commensals against β-lactam antibiotics — Supplementary Data 

# Cephalosporinases associated with outer membrane vesicles released by *Bacteroides* spp. protect gut pathogens and commensals against β-lactam antibiotics

## Supplementary Data

Supplementary Data

**Files in this Data Supplement:**

- Supplementary Table 1 - docx file
