# Supplementary material for: Cephalosporinases associated with outer membrane vesicles released by Bacteroides spp. protect gut pathogens and commensals against β-lactam antibiotics
Source: J Antimicrob Chemother. 2014 Nov 27;70(3):701–9. doi: 10.1093/jac/dku466 (PMC4319488; doi:10.1093/jac/dku466)
Supplement: Supplementary Data [file supp_dku466_dku466supp_table1.docx]

**Supplementary data**

| **Table S1.** Sequence of primers used in this study ^a^ | |
| --- | --- |
| Primer | Sequence (5’→ 3’) |
| BT4507_1 | GACTGAGCTCAGATCGAACATACATAACAG |
| BT4507_2 | GACTGGATCCCAGGATGAATGAGCGCATAC |
| BT4507_3 | GACTGTCGACCATTGCGGACATCTCACGTA |
| BT4507_4 | GACTCTGCAGCACTGCTTCCGACATGTATC |
| Lactamase_F | CGCTCATTCATCCTGCTTC |
| Lactamase_*Eco*RI_R | ATATGAATTCTTATTGATGCGTCACA |
| Reverse | AGCGGATAACAATTTCACACAGGA |
| 20-Lact_R | GGATGAATGAGCGCATGGTGTCTTTTCTTTTATAT |
| 20-Lact_F | ATATAAAAGAAAAGACACCATGCGCTCATTCATCC |
| ^a^ Restriction sites are underlined | |
